# Effect on Quality Characteristics of Tomatoes Grown Under Well-Watered and Drought Stress Conditions

**DOI:** 10.3390/foods6080056

**Published:** 2017-07-25

**Authors:** Warinporn Klunklin, Geoffrey Savage

**Affiliations:** Faculty of Agriculture and Life Sciences, Lincoln University, Lincoln 7647, New Zealand; savage@lincoln.ac.nz

**Keywords:** tomato, drought, water stress, antioxidants, lycopene, antioxidant activities

## Abstract

Tomatoes are one of the most nutritionally and economically important crops in New Zealand and around the world. Tomatoes require large amounts of water to grow well and are adversely affected by drought stress. However, few studies have evaluated the physicochemical characteristics of commercial tomatoes grown under water stress conditions. Four tomato cultivars (Incas, Marmande, Scoresby Dwarf, and Window Box Red) were grown in a greenhouse under well-watered and drought stress conditions and the tomatoes were harvested when ripe. The physicochemical properties and antioxidant contents of the fruits were compared. There were significant differences between cultivars in quality characteristics—such as dry matter, total soluble solids, and pH parameters—but there were no differences in the quality characteristics between the two treatments of the fruits (*p* > 0.05); however, there were significant differences (*p* < 0.05) in the antioxidant compositions (lycopene, total phenolics, and flavonoids) and antioxidant activities (DPPH and ABTS) of the fruits of both cultivars and treatments. Overall, these results indicated that tomatoes increased their bioactive compounds without changing any quality characteristics when exposed to water stress conditions.

## 1. Introduction

Tomatoes (*Solanum lycopersicum* L.) belong to the *Solanaceae* family, which contains about 2800 species and are one of the most important vegetables and economically important crops in New Zealand and around the world [1]. In New Zealand, fresh tomatoes are primarily grown in greenhouses and the tomato fruits are produced almost all year round [2]. Tomatoes are important constituents of human diets; they contain about 94% water, 2.5% total sugars, 2% total fibre, 1% proteins, and other nutritional compounds (acids, lipids, amino acids, and carotenoids) [3]. Tomatoes also contain high levels of other bioactive compounds such as phenolics, vitamin C, and provitamin A, which are thought to protect and possibly prevent cancer [4]. Lycopene is the major carotenoid in the fruit; it accumulates in the final ripening stage of tomatoes as an orange-red pigment and accounts for more than 80% of the total carotenoid content. Lycopene is a fat soluble compound existing as a small globules in the peripheral pericarp and β-carotene is mainly associated with the pectin fraction. Lycopene has strong in vitro and in vivo antioxidant properties [5]. Lycopene and the other active compounds in tomatoes—such as total phenolic contents, ascorbic acid, carotenoids, and total flavonoids—have interested many researchers because of their biological and physicochemical properties, especially their natural antioxidant compounds and human health benefits. Tomatoes are highly sensitive to environmental factors such as temperature, light, and changes in irrigation throughout the growth of the plant [6].

Drought is an important environmental stress at various levels in the plant’s metabolism [7,8]. Approximately one-third of agricultural land in the world experiences an inadequate water supply [8]. Limitation of water supply has an immediate negative impact on the efficient use of water in the plant and it has effects on photosynthesis, plant growth, and production of fruits. Plants respond to water-deficit conditions by disrupting cellular pathways or whole plant functions [6]. Environmental stresses affect both tomato physiology and the synthesis of secondary metabolites such as phenolic acids, avonoids, and terpenoids [9]. Nevertheless, water-deficit may benefit tomato fruit quality due to the increased levels of total soluble solids (sugars, amino acids, and organic acids), which are major compounds which accumulate in the fruit [10,11]. A rise of soluble solids increases the value of the fresh fruits and improves the quality of the fruits because it affects the flavour, taste, and water content of the fruits. In addition, plants growing under stress conditions react by increasing their antioxidant production from both non-enzymatic systems (e.g., flavonoids, phenolic compounds, vitamins C and E, and carotenoids) and enzymatic systems (e.g., superoxide dismutase, glutathione reductase, catalase, and several peroxidases) [12].

The aims of this study were to: (i) characterise the response of different cultivars of tomatoes grown under drought stress; and (ii) investigate the effect of drought stress on the dry matter (DM), total soluble solids (TSS), pH, and antioxidant contents of the fruits that are important from the point of view of fruit quality.

## 2. Materials and Methods

### 2.1. Plant Material and Growing Conditions 

Four different tomato cultivars (cv. Incas, Marmande, Scoresby Dwarf, and Window Box Red) were used in this study. Such varieties are different in shape and size but they are all red fleshed: Incas is a Roma type with plum-shaped fruits; Marmande is a large, juicy beefsteak tomato; Scoresby Dwarf is very useful for commercial growers, producing tomatoes with round red fruits; Window Box Red is a bush type with red cherry tomato fruits. Incas, Marmande, and Window Box Red seeds were purchased from King Seeds Ltd. (Katikati, New Zealand). Scoresby Dwarf seeds were purchased from Bristol Plant and Seeds (Whanganui, New Zealand). The cultivars were arranged in a randomised complete block design with seven replicates for each treatment, giving a total of 14 pots for each cultivar in this trial. Experiments were carried out to measure the response of each cultivar selected under normal watering and water-deficit stress conditions. The plants were maintained between 15–25 °C in a greenhouse in the Horticulture Research Area at Lincoln University, Canterbury, New Zealand (43°38′43″ S, 172°27′43″ E). The tomato seeds were planted in a 3–4 month potting mix. The potting mix was comprised of 80% bark, 20% pumice and Scotts Osmocote^®^ (Scotts Company LLC, Marysville, OH, USA), a controlled release high NPK (Nitrogen, Phosphorous, and Potassium) fertiliser (PGG Wrightson Turf, Christchurch, New Zealand) (16-35-0) containing trace elements (1500 g), horticultural lime (500 g) and hydroflo (500 g) as a wetting agent. Five weeks after germination, single seedling tomatoes were transplanted into individual 5 L plastic pots and allowed to grow in an average temperature of 21 °C (day) and 17 °C (night).

### 2.2. Water Treatments and Harvesting

The plants in the control treatment received water and a nutrient solution (1 part nutrient solution to 100 parts water) at just below the field capacity of the soil (approximately 25% of soil weight) using a time domain reflectometer (Hydrosense^TM^, Campbell Scientific, Inc., Logan, UT, USA) [13]. The drought-treated plants were kept just above wilting by watering them to approximately 10% of soil weight on a 10-day drought cycle [14]. High NK (Nitrogen and Potassium) fertiliser was applied to the potting medium twice, at 12 weeks and 14 weeks, from the start of the experiment. The Window Box Red fruits were harvested after 15 weeks of growth while the Incas, Marmande, and Scoresby Dwarf fruits were harvested after 19 weeks of growth. All undamaged ripe fruits were harvested by hand. Three representative samples were selected randomly from at total of 28 ripe fruits which were harvested from each cultivar and treatment. The harvest was carried out over one day. The harvested fruits were initially stored at −20 °C until further analysis could commence.

### 2.3. Basic Determinations of Tomatoes

The tomatoes were homogenised using a blender (BCG200, Breville Pty Ltd., Sydney, Australia). The dry matter (DM) content was measured by drying overnight at 105 °C using the American Association of Analytical Chemists’ (AOAC) method 935.10 [15]. Tomato juice was squeezed from the fresh tomatoes onto a digital refractometer (PR-100, Atago Co. Ltd., Tokyo, Japan) to measure total soluble solids (TSS) and the results were expressed in °Brix according to AOAC method 932.12 [16]. The pH of the homogenised tomatoes was measured using a pH meter (CH-8603, Mettler-Toledo GmbH, Schwerzenbach, Switzerland). Six replications of each parameter were measured for each sample.

### 2.4. Determination of Antioxidant Compounds 

Lycopene extraction and determination was performed using the method of Sharma and Le Marguer [17]. The total phenolics content of the samples was determined using the method of Jang and Xu [18]. Total flavonoids were extracted by placing the samples in hot ethanol for 1 h and then filtering through a Whatman No. 2 filter paper (Whatman PLC., Maidstone, Kent, UK). All extracted solutions were kept in −20 °C until analysis. The total phenolics were determined using a Folin–Ciocalteu assay [19], and the total flavonoid contents of samples were measured using the aluminium chloride colourimetric method [20]. All analyses were carried out in triplicate.

### 2.5. Determination of Antioxidant Activities (DPPH Activity and ABTS Assay)

Each sample was extracted with a 70% methanol solution. The solutions were then centrifuged at 2500 rpm for 10 min (Rotina 380 R Centrifuge, Hettich Zentrifugen, Tuttlingen, Germany). The supernatants were transferred into individual 10 mL plastic tubes and kept at −20 °C until further analysis could take place. The DPPH (2, 2-diphenyl-2-picrylhydrazyl) radical scavenging activity of the samples was measured using the Mahakunakorn et al. method [21], and the ABTS (2,2′-azinobis-(3-ethylbenzothiazoline-6-sulfonic acid)) radical cation scavenging activity was performed using the method of Re et al. [22].

### 2.6. Data Analysis 

A factorial analysis with four cultivars and two water treatments was applied to assess the differences between treatments in a completely randomised design. All variables were analysed by a two-way ANOVA using SPSS Statistics (version 22.0, SPSS Inc., Chicago, IL, USA) for Mac. Differences between means were established using a least significant differences (LSD) test (*p* < 0.05) using Minitab (version 17, Minitab Inc., State College, PA, USA) for Windows.

## 3. Results

### 3.1. Quality Characteristics Changes in Fruits Induced by Drought Stress

The levels of dry matter, total soluble solids, and pH in the tomatoes grown in a greenhouse under well-watered and water stress conditions are shown in Table 1. There were no significant differences in the DM, TSS, and pH contents between the two treatments. In contrast, there were significant differences in these fruit attributes among the four cultivars across the two water treatments. The Window Box Red fruits had significantly (*p* < 0.05) lower levels of dry matter and total solids, and higher pH values for both treatments, when compared to the other three cultivars.

### 3.2. Antioxidant Compounds 

The overall objective of this experiment was to measure the levels of antioxidant compounds in the flesh of tomato fruits that had either been well-watered or exposed to a 10-day drought cycle while growing. Lycopene, total phenolics, and total flavonoid contents for all cultivars and treatments are shown in Table 2. The lycopene contents of the four cultivars of tomatoes were significantly different (*p* < 0.05) in the well-watered cultivars compared to tomatoes grown under drought conditions. The mean levels of lycopene in the water-deficit fruits were 22.8 mg lycopene/kg DM, in contrast, the well-watered tomatoes were significantly lower (*p* < 0.05). Window Box Red recorded the highest lycopene content when compared to the other three cultivars.

The mean total phenolic contents of the drought-stressed tomatoes were significantly higher than the well-watered tomatoes (46.4 vs. 35.8 mg GAE/100 g DM). There was a significant positive result (*p* < 0.05) for the phenolic contents between the cultivars and treatments. The Window Box Red cultivar showed the highest increase in phenolic contents when exposed to drought conditions compared to the other three cultivars.

There was a significant difference (*p* < 0.05) in the flavonoid contents between the four cultivars of tomatoes. Overall, the mean values showed a small increase between the well-watered and drought-stressed fruits, from 4.9 to 5.0 mg rutin/100 g DM, except for the Incas, which showed a very significant fall in the flavonoid content of the drought-stressed fruits. It is interesting to note that under drought conditions Window Box Red had the highest total flavonoid content which was in contrast to the very low level measured in the well-watered fruits.

### 3.3. Antioxidant Activities of the Fruits

In this study, the DPPH radical scavenging activity and the ABTS free radical scavenging assay was used to evaluate the antioxidant capacity of the fruit tissues (Table 2). The highest DPPH activity in the drought-stressed fruits was detected in Scoresby Dwarf fruits. Window Box Red had the highest ABTS values for both the well-watered and the drought-stressed plants. The mean DPPH values showed a significant difference between the well-watered (1.0 μmol trolox/g DW) and water stress treatments (1.6 μmol trolox/g DW) for all cultivars. The DPPH activity test revealed the highest antioxidant activity changes in the Marmande cultivar when exposed to the water stress treatment.

Tomatoes from different cultivars in both treatments differed significantly (*p* < 0.05) in their ABTS results. The mean ABTS assay results for the four cultivars was 1.8 μmol trolox/g DW for the well-watered fruits, and this was significantly raised to a mean of 3.3 μmol trolox/g DW for the drought-stressed tomatoes. The ABTS assay results for the Incas fruits showed the highest difference assay results between the two treatments.

Overall, the DPPH radical scavenging capacity and the ABTS assay results remained consistently high throughout the study. The DPPH radical scavenging activity and the ABTS free radical scavenging assay of all cultivars showed significantly (*p* < 0.05) higher levels when they had been exposed to drought stress, especially for the Window Box Red cultivar.

## 4. Discussion

Tomato fruit quality can be assessed by the analysis of DM, TSS, and pH [23]. The effect of water-deficit conditions on the growing tomato fruits had no significant effect on their DM, TSS and pH and this confirms a previous study carried out in Florida, USA [24]. Nahar and Gretzmacher [25] reported that a higher DM content was observed in tomatoes exposed to water stress grown in a shade house in Dhaka, Bangladesh. Most of the DM contents in tomatoes are made up from dietary fibre and carbohydrates, which are mainly fructose and glucose [9]. The differences in DM content can affect either the size or yield of tomatoes [26]. Scoresby Dwarf showed the highest DM contents in the well-watered tomatoes (7.8 ± 0.8%) and it can be assumed that these fruits contained higher levels of carbohydrates than the other three cultivars. The results were very close to those reported by Mazur et al. [27] and Sestraş et al. [28] who also showed that the average DM content of cherry tomatoes grown in a greenhouse ranged between 6.6 and 8.0%. Moreover, the DM contents in this study were also similar to the report of local cultivars of tomatoes (a square shaped tomato cultivar) grown in a greenhouse in California, USA giving similar results, after exposure to water stress [29]. However, the chemical compositions of fruits can be altered by drought stress without changing or increasing the DM content [29]. As tomatoes ripen, there is a significant increase in their fructose and glucose contents. These sugars are the largest contributor to the TSS content and, in most cases, the correlation between the TSS and the sugars in the tomatoes is high [30]. In general, soluble solids commonly ranged from 4 to 6 °Brix in the different tomato fruits [31]. The changes in the constituents of the TSS might result from a change in the glucose/fructose ratio and the organic acids in the tomatoes after harvest [32]. Moreover, TSS was reported as a beneficial indicator for the taste of tomatoes [23]. No change in the pH of the fruits was observed; this was similar to the data reported by Alvino et al. [33]. In contrast, the pH of fruits can fall significantly when exposed to drought stress [34,35,36]. The reduction of pH in stressed fruits can be observed during the ripening stages (a colour change in the tomato flesh from pink to red). The acid content of fruits changed because of the reduction of the malic and citric acid contents of the flesh [37].

Lycopene is the most important bioactive compound of tomatoes due to its benefits to human health [38]. The results in this experiment (shown in Table 2) were much higher than the data presented by Shi et al. for mature and firm tomatoes grown in a greenhouse in Ontario, Canada [39], who showed that the mean lycopene content of tomatoes grown in a greenhouse was 75.5 µg/kg DM. Riggi et al. [40] and Atkinson et al. [41] found that drought stress lowed the lycopene content compared to well-watered plants; however, the β-carotene content showed a positive increase. In contrast, Theobald et al. [42] stated that the lycopene contents increased by more than 27% in water-stressed fruits. An increase in lycopene contents was also found in tomato fruits grown in Southern Italy by Favati et al. [43]. Moderate water stress induced an increase of the lycopene concentration of tomatoes [44]. Drought stress initially induces stomatal closure to reduce the effect of water loss by producing a major phytohormone, abscisic acid (ABA). ABA is a primary stress indicator for drought pathways in plants to increase the plants response to desiccation. The lycopene and β-carotene accumulation in the fruits were accompanied by an increase of ABA content [45]. Pek et al. [46] reported that small-fruited tomatoes such as cherry type reached higher lycopene contents than large-fruited cultivars.

In the study of Atkinson et al. [41], the content of total phenolics for the cherry fruits under the water stress conditions was considerably higher than values reported by Barbagallo et al. [9]. Total phenolics were analysed in this study rather than measuring individual polyphenol concentrations in these tomatoes, since no single method can completely identify the polyphenol content of foods due to the structural diversity among the phenolic compounds and the huge variation noticed in different fruits [44]. The Folin–Ciocalteu reagent can also detect other reducing agents such as ascorbic acid, which can interfere with the measurement of the phenolic contents in fruits. The phenolic contents of tomato fruits give the antioxidant capacity in the fruits due to the reduction of oxidative changes in cells by reducing the levels of free radicals [44]. The Window Box Red cultivar has characteristically small fruits that have high total phenolic contents due to their higher skin to volume ratio. Water stress results in the accumulation of antioxidant compounds in the vacuoles of plant dermal tissues and the extracts evaluated in this study were obtained from fruits that were in a mature physiological state [44]. This can also enhance the antioxidant compounds, particularly total flavonoids, because most antioxidant contents are found in the skin [47]. Flavonoids represent a large and diverse group of low molecular weight polyphenolic secondary metabolites in plants, which play an important role in biological processes such as pigmentation of flowers. Rutin is most common flavonoid found in the largest concentration from stalk and tomato fruits such as tomatoes cv. Marmande [48]. Stewart et al. [48] also reported total flavonoids contents of 1.3 ± 22.2 mg rutin/100 g DW in a range of different cultivars imported from different countries and grown in Glasgow, UK, which were similar to the values observed in this study. Water stress produces not only cell-damaging oxidants but also allows the accumulation of a large amount of flavonoids and phenolic acids in the fruits [48]. The various cultivars of tomato respond diversely and generate different amounts of metabolite groups exposed to biotic or abiotic stress [29]. Incas accumulated lower levels of flavonols, quercetin, and kaempferol contents compared to fruits grown in well-watered conditions [46]. The same pattern was also found in irrigated tomato fruits by Pék et al. [46] that well-watered tomato plants produced more rutin in fruits under a controlled environment. Water-deficit conditions can increase the contents of antioxidant compounds—such as lycopene, total phenolics, and total flavonoids—of greenhouse-grown tomato fruits.

In this present study, the ABTS assay gave higher values compared to the DPPH activity as the ABTS assay can react with a wider range of antioxidant compounds. DPPH has been used to determine the in vitro antioxidant activity of extracted tomatoes, which can suggest the potential health benefits of tomato consumption consistent with antioxidant contents, namely lycopene, ascorbic acid, flavonoids and phenolic acids, chlorogenic acids, etc. The DPPH reagent is normally activated by polyphenols (catechins and proanthocyanidins), but does not react with phenolic acids and sugars [49]. Similar low levels of DPPH, ranging from 0.8 to 1.7 μmol trolox/g DW) have also been reported in previous studies [50,51]. Barbagallo et al. [9] also found that the DPPH activity in the fruit of tomato cultivars, Matina and Cochoro, increased under water stress treatments. Quercetin has the highest antioxidative activity using the ABTS assay compared to lycopene, vitamin C, and vitamin E [9]. In addition, previous studies have reported that antioxidant capacities, such as DPPH and ABTS in foods, might differ depending on which solvent is used, the growing season, geographical origin, and agricultural practices [52,53]. This experiment has shown that there were many differences in the chemical and antioxidant compositions of the four different cultivars investigated. The responses of these different cultivars are also very different to water stress from each other.

Overall, the results of this study show that the response of each different cultivar is very different when exposed to different watering regimes.

## 5. Conclusions

This is the first time that a combination of fruit quality characteristics and antioxidant properties of fruits harvested from plants grown under well-watered and drought stress conditions have been evaluated. The quality attributes of the fruits were not decreased by drought; however, the antioxidant compounds and antioxidant capacities showed positive results from this treatment. 

## Figures and Tables

**Table 1:** Chemical characteristics of four cultivars grown under well-watered and drought conditions.

Parameters	Cultivars (C)	Water Treatments (T)		Analysis of Variance			LSD		
		Well-Watered	Drought	C	T	C × T	C	T	C × T
% Dry matter	Scoresby Dwarf	7.8 ± 0.8	7.3 ± 0.7	*	ns	ns	0.4	2.5	3.0
	Incas	7.1 ± 0.5	8.9 ± 0.8						
	Marmande	7.0 ± 0.4	7.4 ± 0.3						
	Window Box Red	6.2 ± 0.5	6.0 ± 0.3						
TSS (°Brix)	Scoresby Dwarf	8.5 ± 0.6	6.5 ± 0.4	*	ns	ns	0.7	2.1	2.7
	Incas	7.5 ± 0.4	7.2 ± 0.7						
	Marmande	7.3 ± 0.9	7.8 ± 0.6						
	Window Box Red	4.7 ± 0.1	4.2 ± 0.1						
pH	Scoresby Dwarf	4.4 ± 0.2	4.5 ± 0.7	*	ns	ns	0.8	1.5	3.0
	Incas	4.7 ± 0.0	4.3 ± 0.2						
	Marmande	4.1 ± 0.1	4.0 ± 0.0						
	Window Box Red	7.0 ± 0.3	5.9 ± 0.2						

Values represent means ± SE (*n* = 6). ns = not significant; * The mean difference is significant at *p* < 0.05, LSD = least significant difference.

**Table 2:** Antioxidant compounds and antioxidant activities (DPPH activity and ABTS assay) evaluated on four tomato cultivars grown under well-watered and drought conditions.

Parameters	Cultivars (C)	Water Treatments (T)		Analysis of Variance			LSD		
		Well-Watered	Drought	C	T	C × T	C	T	C × T
Lycopene (mg/kg DW)	Scoresby Dwarf	21.1 ± 0.7	22.8 ± 0.5	*	*	*	0.7	1.0	1.9
	Incas	20.1 ± 0.2	21.4 ± 0.7						
	Marmande	18.8 ± 0.4	20.6 ± 0.3						
	Window Box Red	22.2 ± 0.4	26.3 ± 0.2						
Total phenolics (mg GAE/100 g DW)	Scoresby Dwarf	38.0 ± 0.6	48.6 ± 0.5	*	*	*	0.9	1.2	2.3
	Incas	34.3 ± 0.6	46.9 ± 0.4						
	Marmande	32.7 ± 0.8	40.9 ± 0.4						
	Window Box Red	38.2 ± 0.3	49.3 ± 0.9						
Flavonoids (mg rutin/100 g DW)	Scoresby Dwarf	5.2 ± 0.1	6.1 ± 0.1	*	*	*	0.1	0.2	0.4
	Incas	6.7 ± 0.0	1.7 ± 0.1						
	Marmande	4.8 ± 0.1	5.1 ± 0.1						
	Window Box Red	2.9 ± 0.1	6.9 ± 0.1						
DPPH (μmol trolox/g DW)	Scoresby Dwarf	1.0 ± 0.1	1.8 ± 0.0	*	*	ns	0.2	0.3	1.1
	Incas	0.8 ± 0.0	1.3 ± 0.1						
	Marmande	0.7 ± 0.0	1.4 ± 0.0						
	Window Box Red	1.3 ± 0.0	1.7 ± 0.0						
ABTS (μmol trolox/g DW)	Scoresby Dwarf	1.4 ± 0.1	3.2 ± 0.1	*	*	*	0.2	0.2	1.3
	Incas	0.7 ± 0.10	2.6 ± 0.1						
	Marmande	0.7 ± 0.10	2.3± 0.1						
	Window Box Red	4.6 ± 0.07	5.1 ± 0.1						

Values represent mean ± standard deviation (*n* = 3). ns = not significant; * The mean difference is significant at *p* < 0.05, LSD = least significant difference.
